# A Novel Extension of the Technique for Order Preference by Similarity to Ideal Solution Method with Objective Criteria Weights for Group Decision Making with Interval Numbers

**DOI:** 10.3390/e23111460

**Published:** 2021-11-03

**Authors:** Dariusz Kacprzak

**Affiliations:** Department of Mathematics, Faculty of Computer Science, Bialystok University of Technology, Wiejska 45A, 15-351 Bialystok, Poland; d.kacprzak@pb.edu.pl

**Keywords:** interval numbers, MCGDM, TOPSIS, entropy, objective weights

## Abstract

This paper presents an extension of the Technique for Order Preference by Similarity to Ideal Solution (TOPSIS) method with objective criteria weights for Group Decision Making (GDM) with Interval Numbers (INs). The proposed method is an alternative to popular and often used methods that aggregate the decision matrices provided by the decision makers (DMs) into a single group matrix, which is the basis for determining objective criteria weights and ranking the alternatives. It does not use an aggregation operator, but a transformation of the decision matrices into criteria matrices, in the case of determining objective criteria weights, and into alternative matrices, in the case of the ranking of alternatives. This ensures that all the decision makers’ evaluations are taken into account instead of their certain average. The numerical example shows the ease of use of the proposed method, which can be implemented into common data analysis software such as Excel.

## 1. Introduction

Recent years show that Multiple Criteria Decision Making (MCDM) methods are increasingly used to solve real decision-making problems concerning various aspects of human life [1,2,3]. The main application areas for these methods are supply chain management [4], logistics [5], engineering [6], technology [7], and many others. The complexity and diversity of MCDM problems have resulted in the development of a variety of methods to solve them [2]. One group of these methods are methods based on reference points. Historically, the first method which belongs to this group is the Hellwig method [8]. It uses a single reference point, called a “pattern”. It is an artificial solution that maximizes benefit criteria and minimizes cost criteria. The computed synthetic indicator “proximity” of the alternatives to the “pattern” allows for their linear ordering and the identification of the best one. However, the most recognized and regularly used method in this group is TOPSIS, developed by Hwang and Yoon [9]. It uses two artificial solutions called the Positive Ideal Solution (PIS) and the Negative Ideal Solution (NIS). The PIS is equivalent to the “pattern” in Hellwig’s method. In turn, the NIS minimizes the benefit criteria and maximizes the cost criteria. Taking into account the separation of the alternatives from the PIS and NIS, the Relative Closeness Coefficients (RCCs) to the PIS are calculated, which allows for the ranking of the alternatives.

The applications of the TOPSIS method are very diverse. Apart from the main applications of MCDM mentioned above, it is used in more and more new areas, such as flow control in a manufacturing system [10], the selection of sustainable acid rain control options [11], the selection of the best employees using decision support systems in internal control [12], credit risk evaluations for strategic partners [13], the investigation of aggregated social influence [14], the selection of stocks before the formation of a portfolio based on a company’s financial performance [15], the identification of the best wind turbines for different locations [16], the ranking of the developmental performance of nations [17], the evaluation of the quality of institutions in the European Union countries [18], the evaluation of technologies improving the quality of life of elderly people [19], and many others.

In real-life problems, it may be difficult to measure data accurately or present the preferences of the DMs by real numbers; it may also happen that DMs use linguistic variables, in which case we can use another format of data. In such situations, MCDM methods, including TOPSIS, should be extended from real numbers to the new type of data. In the literature, we can find a number of extensions of the TOPSIS method for different types of data: fuzzy numbers [20], ordered fuzzy numbers [21], hesitant fuzzy sets [22], intuitionistic fuzzy sets [23], hesitant Pythagorean fuzzy sets [24], interval-valued fuzzy sets [25], interval neutrosophic sets [26], and others. This shows that researchers are developing new ways of presenting data to allow DMs to formulate their preferences more effectively. We can say that the choice of a data presentation method is an MCDM problem.

In this paper we use INs. An extension of the TOPSIS method to MCDM problems with INs was developed by Jahanshahloo et al. [27]. A limitation of this approach is the definitions of the PIS and NIS. These reference points are represented by real numbers selected from the lower and upper endpoints of the INs in the decision matrix, rather than by INs themselves. This can lead to incorrect results [28]. In the literature, various methods for determining the PIS and NIS for INs have been proposed. In [29,30], they are represented by real numbers instead of intervals, as in [27]. In [31,32], the PIS is defined as an interval whose endpoints are the maximum values from the lower and upper endpoints of the intervals, respectively, while for the NIS we take the minimum values of these endpoints. In [33], the PIS is the average of intervals, while for the NIS, the lower endpoints are the minimum of the lower endpoints of the intervals and the upper endpoints are the maximum of the upper endpoints of the intervals, respectively. The main limitation of these methods is that the determined elements of the PIS and NIS may not be elements of the decision matrix. Dymova et al. [28] presented a method of comparing INs to determine the minimum and maximum elements from the decision matrix. It is based on determining the distance between the midpoints of the INs being compared. In the proposed approach, we will use an analogous method of comparing INs, as proposed by Hu and Wang [34].

An important step in MCDM methods, including the TOPSIS method, is the determination of criteria weights. These describe the importance of each criterion in the decision-making process and have a key influence on the final result. We usually use subjective or objective weights in solving MCDM problems. Subjective weights are determined by the DM or an expert, using their knowledge, experience, skills, etc. In situations where we cannot obtain the appropriate weights or the cost of obtaining them is too high, we can use objective weights. These are determined by using mathematical methods based on the decision matrix. One of the popular methods for determining objective weights is the entropy method [9]. It assigns a higher weight to the given criterion, regarding which the evaluations of alternatives are more diversified. Hosseinzadeh Lotfi and Fallahnejad [35] proposed an extension of the entropy method to data in the form of INs. As a result, we can obtain objective criteria weights, also in the form of INs.

Because of the increasing complexity of decision-making problems, they are often analyzed by a group of DMs, which leads to the development of so-called Multiple Criteria Group Decision Making (MCGDM). In such situations, each member of the group defines an individual decision matrix. A common technique is to determine the aggregate (group) matrix from the individual matrices using a selected aggregation operator. This matrix is the basis for determining objective criteria weights and ranking the alternatives. One of the most popular aggregation operators is the arithmetic mean. Note, however, that this may not reflect the preferences or judgments of DMs [36]. To better explain this limitation, we present two simple numerical examples. We consider a group of two decision makers $\left\{DM_{1},DM_{2}\right\}$ who evaluate three alternatives $\left\{A_{1},\;A_{2},A_{3}\right\}$ with respect to two benefit criteria $\left\{C_{1},C_{2}\right\}$ using the following scale: {1,2,3,4,5}. Their evaluations of the alternatives with respect to the criteria are in the form of individual decision matrices $X_{1}$ and $X_{2}$; by $X_{ART}$ we denote the aggregation results using the arithmetic mean.

**Example** **1.** *The ratings of the alternatives with respect to the criteria provided by the DMs are:*

$$X_{1}=\begin{array}{cc} DM_{1} & \begin{array}{cc} C_{1} & C_{2} \end{array}\\ \begin{array}{c} A_{1}\\ A_{2}\\ A_{3} \end{array} & \left(\begin{array}{cc} 1 & 1\\ 2 & 2\\ 4 & 3 \end{array}\right) \end{array},\;\;\;\;X_{2}=\begin{array}{cc} DM_{2} & \begin{array}{cc} C_{1} & C_{2} \end{array}\\ \begin{array}{c} A_{1}\\ A_{2}\\ A_{3} \end{array} & \left(\begin{array}{cc} 3 & 3\\ 2 & 2\\ 1 & 1 \end{array}\right) \end{array}\;.$$

*Let us note that regardless of whether the ratings of the alternatives with respect to a criterion are in the form “1 and 3”, “2 and 2”, or “3 and 1”, the aggregation results are the same and equal to “2”. The aggregation results are:*

$$X_{AGG}=\begin{array}{cc} & \begin{array}{cc} C_{1} & C_{2} \end{array}\\ \begin{array}{c} A_{1}\\ A_{2}\\ A_{3} \end{array} & \left(\begin{array}{cc} 2 & 2\\ 2 & 2\\ 2.5 & 2 \end{array}\right) \end{array}\;.$$

*Based on matrix* $X_{AGG}$, *and using the entropy method, we can calculate the criteria weights, obtaining the following vector:*

$$w_{AGG}=\left(1,\;0\right)\;.$$

*This means that criterion* $C_{2}$ *has no influence on the ranking of the alternatives and can be omitted. On the other hand, using the proposed approach to the matrices* $X_{1}$ *and* $X_{2}$, *we obtain the following vector of criteria weights:*

w=(0.5921, 0.4079) .

**Example** **2.** *The ratings of the alternatives with respect to the criteria provided by the DMs are:*

$$X_{1}=\begin{array}{cc} DM_{1} & \begin{array}{cc} C_{1} & C_{2} \end{array}\\ \begin{array}{c} A_{1}\\ A_{2}\\ A_{3} \end{array} & \left(\begin{array}{cc} 5 & 1\\ 3 & 2\\ 1 & 3 \end{array}\right) \end{array},\;\;\;X_{2}=\begin{array}{cc} DM_{2} & \begin{array}{cc} C_{1} & C_{2} \end{array}\\ \begin{array}{c} A_{1}\\ A_{2}\\ A_{3} \end{array} & \left(\begin{array}{cc} 1 & 3\\ 3 & 2\\ 5 & 1 \end{array}\right) \end{array}\;.$$

*The aggregation results are:*

$$X_{AGG}=\begin{array}{cc} & \begin{array}{cc} C_{1} & C_{2} \end{array}\\ \begin{array}{c} A_{1}\\ A_{2}\\ A_{3} \end{array} & \left(\begin{array}{cc} 3 & 2\\ 3 & 2\\ 3 & 2 \end{array}\right) \end{array}\;.$$

*Matrix* $X_{AGG}$ *shows that all three alternatives*$\left\{A_{1},\;A_{2},A_{3}\right\}$ *are equivalent (i.e., they have the same aggregate rating) and we cannot calculate the vector of criteria weights using the entropy method. However, if we use the proposed approach, we obtain the following vector of criteria weights:*

w=(0.6497, 0.3503) .

From Examples 1 and 2, we can conclude that such an averaged result does not reflect the discrepancies between the individual decisions (the preferences of the DMs) and the fact that using such averaged information may lead to an incorrect final decision.The aim of this paper is to present a new approach for GDM using the TOPSIS method and objective criteria weights with INs. The first main contribution of this paper is a method for determining the objective criteria weights for GDM without aggregating individual decision matrices. The method involves transforming the individual decision matrices into criteria matrices and using the interval entropy and the interval TOPSIS methods to determine the objective criteria weights. In this method, unlike in the method proposed by Hosseinzadeh Lotfi and Fallahnejad [35], as the final result, we receive the weights in the form of real numbers. The second main contribution of this paper is the TOPSIS method for GDM, also without the aggregation of individual decision matrices. This method involves transforming the decision matrices into matrices of alternatives and then using a new interval TOPSIS method for the ranking of alternatives.

The remainder of the paper consists of the following sections. Section 2 presents basic information about INs and a description of the classical TOPSIS method and the classical entropy method. The main section of the paper, i.e., Section 3, presents the algorithm of the proposed method in detail. Next, the proposed method is used in a numerical example and compared with other, similar approaches which are based on the aggregation of individual matrices. The paper ends with the conclusions.

## 2. Preliminaries

In the following, we present some basic information about INs, the classical TOPSIS method, and the entropy method of determining criteria weights.

### 2.1. Interval Numbers

**Definition** **1.** *As proposed by [37]: The closed IN, denoted by* $\left[\underline{a},\bar{a}\right]$*, is the set of real numbers given by:*

(1) $$\left[\underline{a},\bar{a}\right]=\left\{x\in \mathbb{R}\;:\;\underline{a}\leq x\leq \bar{a}\right\}\;.$$

Throughout this paper, INs will be used in the interval TOPSIS and interval entropy methods, so we assume that they are positive INs, i.e., $\underline{a}>0$.

**Definition** **2.** *As proposed by [37]: Let* $\left[\underline{a},\bar{a}\right]$ *and* $\left[\underline{b},\bar{b}\right]$ *be two positive INs, and* λ>0 *be a real number. Then:*

$$\begin{array}{c} \left[\underline{a},\bar{a}\right]=\left[\underline{b},\bar{b}\right]\;if\;\underline{a}=\underline{b}\;and\;\bar{a}=\bar{b},\\ \left[\underline{a},\bar{a}\right]+\left[\underline{b},\bar{b}\right]=\left[\underline{a}+\underline{b},\;\bar{a}+\bar{b}\right],\\ \left[\underline{a},\bar{a}\right]-\left[\underline{b},\bar{b}\right]=\left[\underline{a}-\bar{b},\;\bar{a}-\underline{b}\right],\\ \left[\underline{a},\bar{a}\right]\cdot \left[\underline{b},\bar{b}\right]=\left[\underline{a}\cdot \underline{b},\;\bar{a}\cdot \bar{b}\right],\\ \left[\underline{a},\bar{a}\right]/\left[\underline{b},\bar{b}\right]=\left[\underline{a}/\bar{b},\;\bar{a}/\underline{b}\right],\\ \lambda \cdot \left[\underline{a},\bar{a}\right]=\left[\lambda \cdot \underline{a},\lambda \cdot \bar{a}\right]\;.\; \end{array}$$

The TOPSIS method requires the determination of the minimum and maximum elements. To compare INs, we apply the method developed by Hu and Wang [34]. It is based on a different description of INs than Equation (1) used in Definition 1.

**Definition** **3.** *As proposed by [34]: The IN* $\left[\underline{a},\bar{a}\right]$ *is represented in the form:*

(2) $$\langle m\left(\left[\underline{a},\bar{a}\right]\right);w\left(\left[\underline{a},\bar{a}\right]\right)\rangle$$

*where* $m\left(\left[\underline{a},\bar{a}\right]\right)$ *and* $w\left(\left[\underline{a},\bar{a}\right]\right)$ *are its mid-point and half-width, respectively, determined as follows:*

(3) $$m\left(\left[\underline{a},\bar{a}\right]\right)=\frac{\underline{a}+\bar{a}}{2}\;,$$

*and:*

(4) $$w\left(\left[\underline{a},\bar{a}\right]\right)=\frac{\bar{a}-\underline{a}}{2}\;.$$

Using the representation from Equation (2), Hu and Wang defined the order relation “${≺}_{=}$” for INs as follows.

**Definition** **4.** *As proposed by [34]: Let* $\left[\underline{a},\bar{a}\right]$ *and* $\left[\underline{b},\bar{b}\right]$ *be two INs. Then:*

(5) $$\left[\underline{a},\bar{a}\right]{≺}_{=}\left[\underline{b},\bar{b}\right]\;iff\;\{\begin{array}{ccc} m\left(\left[\underline{a},\bar{a}\right]\right)<m\left(\left[\underline{b},\bar{b}\right]\right), & if & m\left(\left[\underline{a},\bar{a}\right]\right)\neq m\left(\left[\underline{b},\bar{b}\right]\right)\\ w\left(\left[\underline{a},\bar{a}\right]\right)\geq w\left(\left[\underline{b},\bar{b}\right]\right), & if & m\left(\left[\underline{a},\bar{a}\right]\right)=m\left(\left[\underline{b},\bar{b}\right]\right) \end{array}.$$

*and:*

(6) $$\left[\underline{a},\bar{a}\right]≺\left[\underline{b},\bar{b}\right]\;iff\;\left[\underline{a},\bar{a}\right]{≺}_{=}\left[\underline{b},\bar{b}\right]\;and\;\left[\underline{a},\bar{a}\right]\neq \left[\underline{b},\bar{b}\right].$$

### 2.2. The Classical TOPSIS Method

Suppose an MCDM problem is given. The solution of the problem involves the linear ordering of the set of possible alternatives $\left\{A_{1},A_{2},\ldots ,\;A_{m}\right\}$ and the indication of the best one. The alternatives under consideration are evaluated with respect to a set of criteria $\left\{C_{1},C_{2},\ldots ,\;C_{n}\right\}$ that determine the choice of a solution. An MCDM problem is represented by a decision matrix X, of the form:

(7) $$X=\left(\begin{array}{cc} \begin{array}{cc} x_{11} & x_{12}\\ x_{21} & x_{22} \end{array} & \begin{array}{cc} \cdots & x_{1n}\\ \cdots & x_{2n} \end{array}\\ \begin{array}{cc} \vdots & \vdots \\ x_{m1} & x_{m2} \end{array} & \begin{array}{cc} \ddots & \vdots \\ \cdots & x_{mn} \end{array} \end{array}\right)$$

where $x_{ij}$ for i=1,2,…,m and j=1,2,…,n represents the evaluation of the ith alternative with respect to the jth criterion. In addition, we determine the vector criteria weights $w=\left(w_{1},w_{2},\ldots ,w_{n}\right)$. The classical TOPSIS method developed by Hwang and Yoon consists of the following steps [9]:

**Step 1.** The normalization of the decision matrix X and calculation of the matrix Y, of the form:
  (8) $$Y=\left(\begin{array}{cc} \begin{array}{cc} y_{11} & y_{12}\\ y_{21} & y_{22} \end{array} & \begin{array}{cc} \cdots & y_{1n}\\ \cdots & y_{2n} \end{array}\\ \begin{array}{cc} \vdots & \vdots \\ y_{m1} & y_{m2} \end{array} & \begin{array}{cc} \ddots & \vdots \\ \cdots & y_{mn} \end{array} \end{array}\right)$$
  using, for j=1,..,n, the following formula:
  (9) $$y_{ij}=\frac{x_{ij}}{\sqrt{\sum_{i=1}^{m}x_{ij}^{2}}}\;.$$
**Step 2.** The calculation of the weighted normalized decision matrix V, of the form:
  (10) $$V=\left(\begin{array}{cc} \begin{array}{cc} v_{11} & v_{12}\\ v_{21} & v_{22} \end{array} & \begin{array}{cc} \cdots & v_{1n}\\ \cdots & v_{2n} \end{array}\\ \begin{array}{cc} \vdots & \vdots \\ v_{m1} & v_{m2} \end{array} & \begin{array}{cc} \ddots & \vdots \\ \cdots & v_{mn} \end{array} \end{array}\right)$$
  where $v_{ij}=w_{j}\cdot y_{ij}$ for i=1,2,…,m and j=1,2,…,n.
**Step 3.** Determination of the PIS $\left(A^{+}\right)$, of the form:
  (11) $$A^{+}=\left(v_{1}^{+},v_{2}^{+},\ldots ,v_{n}^{+}\right)=\left\{\left(\underset{i}{\max}v_{ij}\;|\;j\in B\right),\left(\underset{i}{\min}v_{ij}\;|\;j\in C\right)\right\},$$
  and of the NIS $\left(A^{-}\right)$, of the form:
  (12) $$A^{-}=\left(v_{1}^{-},v_{2}^{-},\ldots ,v_{n}^{-}\right)=\left\{\left(\underset{i}{\min}v_{ij}\;|\;j\in B\right),\left(\underset{i}{\max}v_{ij}\;|\;j\in C\right)\right\},$$
  where B and C are associated with benefit and cost criteria, respectively.
**Step 4.** The calculation of the distance of each $A_{i}$ (i=1,…,m) from the PIS:
  (13) $$d_{i}^{+}=\sqrt{\sum_{j=1}^{n}{\left(v_{ij}-v_{j}^{+}\right)}^{2}},$$
  and from the NIS:
  (14) $$d_{i}^{-}=\sqrt{\sum_{j=1}^{n}{\left(v_{ij}-v_{j}^{-}\right)}^{2}}.$$
**Step 5.** The calculation of the coefficients $RCC_{i}$ (i=1, 2,…,m) of relative closeness to the PIS for each alternative $A_{i}$ (i=1,…,m), using the following formula:
  (15) $$RCC_{i}=\frac{d_{i}^{-}}{d_{i}^{+}+d_{i}^{-}}.$$
**Step 6.** The ranking of alternatives in descending order, using $RCC_{i}$, and the determination of the best one (the one with the highest value of $RCC_{i}$).

### 2.3. The Entropy Method 

The starting point for determining objective criteria weights by the entropy method is the decision matrix, Equation (7) (see Section 2.2). It consists of the following steps [9]:

**Step 1.** The normalization of the decision matrix X and the calculation of the matrix Y, of the form:
  (16) $$Y=\left(\begin{array}{cc} \begin{array}{cc} y_{11} & y_{12}\\ y_{21} & y_{22} \end{array} & \begin{array}{cc} \cdots & y_{1n}\\ \cdots & y_{2n} \end{array}\\ \begin{array}{cc} \vdots & \vdots \\ y_{m1} & y_{m2} \end{array} & \begin{array}{cc} \ddots & \vdots \\ \cdots & y_{mn} \end{array} \end{array}\right)$$
  using the following formula for j=1,..,n:
  (17) $$y_{ij}=\frac{x_{ij}}{\sum_{i=1}^{m}x_{ij}}.$$
**Step 2.** The calculation of the vector of entropy $e=\left(e_{1},e_{2},\ldots ,e_{n}\right)$, using the following formula for j=1,…,n:
  (18) $$e_{j}=-\frac{1}{\ln m}\sum_{i=1}^{m}y_{ij}\ln y_{ij}.$$
  Moreover, when $y_{ij}=0$ for some i, the value of $y_{ij}\ln y_{ij}$ is taken as 0, which is consistent with $\underset{x\to 0^{+}}{\lim}x\ln x=0$.
**Step 3.** The calculation of the vector of diversification $d=\left(d_{1},d_{2},\ldots ,d_{n}\right)$, using the following formula for j=1,…,n:
  (19) $$d_{j}=1-e_{j}.$$
**Step 4.** The calculation of the vector of objective criteria weights $w=\left(w_{1},w_{2},\ldots ,w_{n}\right)$, where:
  (20) $$w_{j}=\frac{d_{j}}{\sum_{j=1}^{n}d_{j}}.$$

## 3. The Proposed Approach

The proposed extension of the TOPSIS method with objective criteria weights based on interval data for GDM consists of three major stages:

- The preparation of the data;
- The calculation of the objective criteria weights using the interval entropy method and the interval TOPSIS method, without the aggregation of individual decision matrices;
- The linear ordering of alternatives using the extended TOPSIS method, based on interval data, without the aggregation of individual decision matrices.

**Figure 1:**
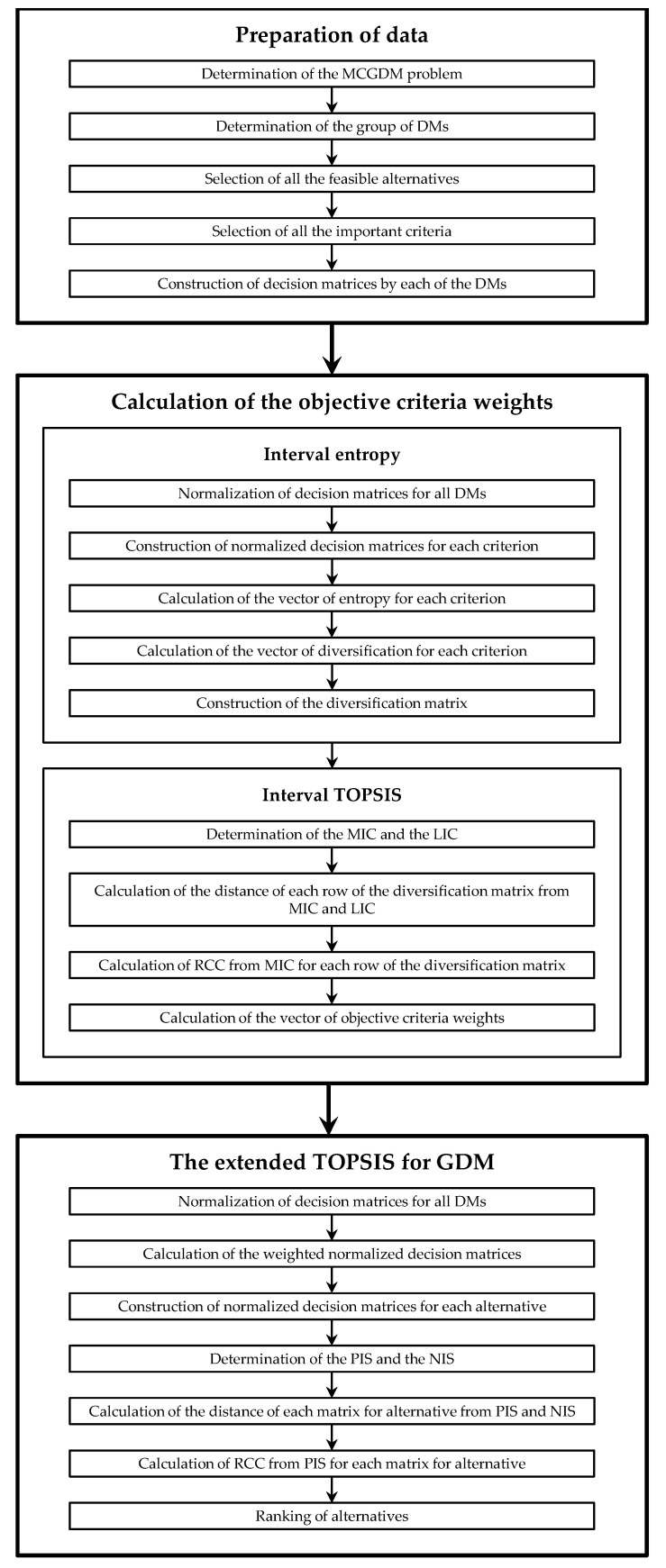
The conceptual framework of the proposed method.

**Figure 2:**
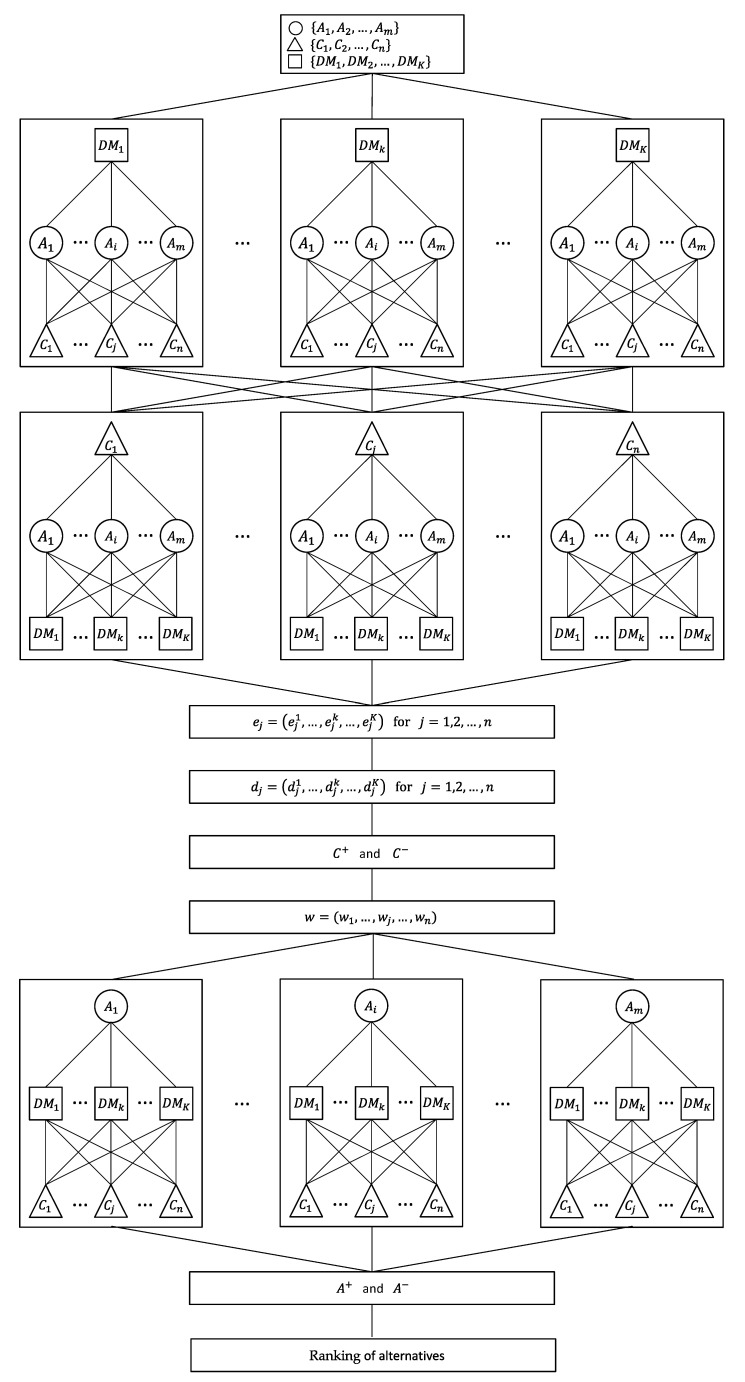
Hierarchical structure of the proposed method.

A flow chart and a graphical scheme of the proposed method are shown in Figure 1 and Figure 2, respectively.

**Stage 1:** The preparation of the data.
  As in Section 2.2., suppose an MCDM problem for GDM is given, which consists of a set of possible alternatives $\left\{A_{1},A_{2},\ldots ,\;A_{m}\right\}$ and a set of criteria $\left\{C_{1},C_{2},\ldots ,\;C_{n}\right\}$. In this case, the evaluation of alternatives, with respect to the criteria, is performed by a group of DMs or experts $\left\{DM_{1},DM_{2},\ldots ,\;DM_{K}\right\}$. In the process of GDM, each $DM_{k}$ (k=1, 2,…,K) constructs a matrix, called the individual decision matrix, of the form:
  (21) $$X^{k}=\begin{array}{cc} DM_{k} & \begin{array}{cc} \begin{array}{cc} C_{1}\; & \;C_{2}\;\;\; \end{array} & \begin{array}{cc} \cdots & C_{n} \end{array} \end{array}\\ \begin{array}{c} \begin{array}{c} A_{1}\\ A_{2} \end{array}\\ \begin{array}{c} \vdots \\ A_{m} \end{array} \end{array} & \left(\begin{array}{cc} \begin{array}{cc} x_{11}^{k} & x_{12}^{k}\\ x_{21}^{k} & x_{22}^{k} \end{array} & \begin{array}{cc} \cdots & x_{1n}^{k}\\ \cdots & x_{2n}^{k} \end{array}\\ \begin{array}{cc} \vdots & \vdots \\ x_{m1}^{k} & x_{m2}^{k} \end{array} & \begin{array}{cc} \ddots & \vdots \\ \cdots & x_{mn}^{k} \end{array} \end{array}\right) \end{array}.$$
  In the proposed approach, each element $x_{ij}^{k}$ for i=1,2,…,m and j=1,2,…,n of the matrix $X^{k}$ is in the form of an IN, i.e., $x_{ij}^{k}=\left[{\underline{x}}_{ij}^{k},{\bar{x}}_{ij}^{k}\right]$, and represents the evaluation of the kth DM of the ith alternative with respect to the jth criterion.
**Stage 2:** The calculation of the objective criteria weights for GDM, without the aggregation of individual decision matrices.
  The proposed method of calculation of the objective criteria weights based on interval entropy and interval TOPSIS consists of the following steps.

**Step 1.** The normalization, for each decision maker $DM_{k}$ (k=1,2,…,K), of their individual decision matrix, as given by Equation (21), and obtaining the matrix $Y^{k}$, of the form:
  (22) $$Y^{k}=\begin{array}{cc} DM_{k} & \begin{array}{cc} \begin{array}{cc} C_{1} & \;\;C_{2}\;\; \end{array} & \begin{array}{cc} \cdots & \;C_{n} \end{array} \end{array}\\ \begin{array}{c} \begin{array}{c} A_{1}\\ A_{2} \end{array}\\ \begin{array}{c} \vdots \\ A_{m} \end{array} \end{array} & \left(\begin{array}{cc} \begin{array}{cc} y_{11}^{k} & y_{12}^{k}\\ y_{21}^{k} & y_{22}^{k} \end{array} & \begin{array}{cc} \cdots & y_{1n}^{k}\\ \cdots & y_{2n}^{k} \end{array}\\ \begin{array}{cc} \vdots & \vdots \\ y_{m1}^{k} & y_{m2}^{k} \end{array} & \begin{array}{cc} \ddots & \vdots \\ \cdots & y_{mn}^{k} \end{array} \end{array}\right) \end{array}$$
  using the following formula for j=1,..,n [35]:
  (23) $$y_{ij}^{k}=\{\begin{array}{ccc} \left[\frac{{\underline{x}}_{ij}^{k}}{\sum_{i=1}^{m}{\bar{x}}_{ij}^{k}},\frac{{\bar{x}}_{ij}^{k}}{\sum_{i=1}^{m}{\bar{x}}_{ij}^{k}}\right] & \mathrm{if} & j\in B\\ \left[\frac{1/{\bar{x}}_{ij}^{k}}{\sum_{i=1}^{m}1/{\underline{x}}_{ij}^{k}},\frac{1/{\underline{x}}_{ij}^{k}}{\sum_{i=1}^{m}1/{\underline{x}}_{ij}^{k}}\right] & \mathrm{if} & j\in C \end{array}.$$
**Step 2.** The construction, for each criterion $C_{j}$ (j=1,2,…,n), of the matrix $V^{j}$, of the form:
  (24) $$V^{j}=\begin{array}{cc} C_{j} & \begin{array}{cc} \begin{array}{cc} DM_{1} & DM_{2} \end{array} & \begin{array}{cc} \cdots & DM_{K} \end{array} \end{array}\\ \begin{array}{c} \begin{array}{c} A_{1}\\ A_{2} \end{array}\\ \begin{array}{c} \vdots \\ A_{m} \end{array} \end{array} & \left(\begin{array}{cc} \begin{array}{cc} y_{1j}^{1} & y_{1j}^{2}\\ y_{2j}^{1} & y_{2j}^{2} \end{array} & \begin{array}{cc} \cdots & y_{1j}^{K}\\ \cdots & y_{2j}^{K} \end{array}\\ \begin{array}{cc} \vdots & \vdots \\ y_{mj}^{1} & y_{mj}^{2} \end{array} & \begin{array}{cc} \ddots & \vdots \\ \cdots & y_{mj}^{K} \end{array} \end{array}\right) \end{array}.$$
**Step 3.** The calculation, for each criterion $C_{j}$ (j=1,2,…,n), of the entropy vector $e_{j}$, of the form:
  (25) $$e_{j}=\left(e_{j}^{1},e_{j}^{2},\ldots ,e_{j}^{K}\right)$$
  based on the matrix $V^{j}$, where $e_{j}^{k}=\left[{\underline{e}}_{j}^{k},{\bar{e}}_{j}^{k}\right]$ for k=1,2,…,K and:
  (26) $${\underline{e}}_{j}^{k}=\min\left\{-\frac{1}{\ln m}\sum_{i=1}^{m}{\underline{y}}_{ij}^{k}\ln{\underline{y}}_{ij}^{k},-\frac{1}{\ln m}\sum_{i=1}^{m}{\bar{y}}_{ij}^{k}\ln{\bar{y}}_{ij}^{k}\right\},$$
  and:
  (27) $${\bar{e}}_{j}^{k}=\max\left\{-\frac{1}{\ln m}\sum_{i=1}^{m}{\underline{y}}_{ij}^{k}\ln{\underline{y}}_{ij}^{k},-\frac{1}{\ln m}\sum_{i=1}^{m}{\bar{y}}_{ij}^{k}\ln{\bar{y}}_{ij}^{k}\right\},$$
  and ${\underline{y}}_{ij}^{k}\ln{\underline{y}}_{ij}^{k}$ or ${\bar{y}}_{ij}^{k}\ln{\bar{y}}_{ij}^{k}$ is defined to be 0 if ${\underline{y}}_{ij}^{k}=0$ or ${\bar{y}}_{ij}^{k}=0$ [35], respectively.
**Step 4.** The calculation, for each criterion $C_{j}$ (j=1,2,…,n), of the diversification vector $d_{j}$, of the form:
  (28) $$d_{j}=\left(d_{j}^{1},d_{j}^{2},\ldots ,d_{j}^{K}\right)$$
  where $d_{j}^{k}=1-e_{j}^{k}=\left[1-{\bar{e}}_{j}^{k},1-{\underline{e}}_{j}^{k}\right]$ for k=1, 2,…,K, and the construction of diversification matrix D, of the form:
  (29) $$D=\begin{array}{cc} & \begin{array}{cc} \begin{array}{cc} DM_{1} & DM_{2} \end{array} & \begin{array}{cc} \cdots & DM_{K} \end{array} \end{array}\\ \begin{array}{c} \begin{array}{c} C_{1}\\ C_{2} \end{array}\\ \begin{array}{c} \vdots \\ C_{n} \end{array} \end{array} & \left(\begin{array}{cc} \begin{array}{cc} \;d_{1}^{1}\;\; & \;d_{1}^{2}\;\\ \;d_{2}^{1}\;\; & \;d_{2}^{2}\; \end{array} & \begin{array}{cc} \;\cdots & \;d_{1}^{K}\;\\ \;\cdots & \;d_{2}^{K}\; \end{array}\\ \begin{array}{cc} \vdots & \vdots \\ \;d_{n}^{1}\; & \;d_{n}^{2}\; \end{array} & \begin{array}{cc} \ddots & \vdots \\ \;\;\cdots & \;d_{n}^{K}\; \end{array} \end{array}\right) \end{array}.$$
**Step 5.** The determination of the Most Important Criterion (MIC):
  (30) $$C^{+}=\left(c_{1}^{+},c_{2}^{+},\ldots ,c_{K}^{+}\right)$$
  where $c_{k}^{+}=\underset{j}{\max}d_{j}^{k}$ for k=1,2,…,K, and of the Least Important Criterion (LIC):
  (31) $$C^{-}=\left(c_{1}^{-},c_{2}^{-},\ldots ,c_{K}^{-}\right)$$
  where $c_{k}^{-}=\left[0,0\right]$ for k=1,2,…,K, based on the matrix D.
**Step 6.** The calculation of the distance of each diversification vector $d_{j}$, representing the weight of criterion $C_{j}$ (j=1, 2,…,n), from the MIC:
  (32) $$d_{j}^{C+}=\sqrt{\sum_{k=1}^{K}\left[{\left({\underline{d}}_{j}^{k}-{\underline{c}}_{k}^{+}\right)}^{2}+{\left({\bar{d}}_{j}^{k}-{\bar{c}}_{k}^{+}\right)}^{2}\right]},$$
  and from the LIC:
  (33) $$d_{j}^{C-}=\sqrt{\sum_{k=1}^{K}\left[{\left({\underline{d}}_{j}^{k}-{\underline{c}}_{k}^{-}\right)}^{2}+{\left({\bar{d}}_{j}^{k}-{\bar{c}}_{k}^{-}\right)}^{2}\right]}.$$
**Step 7.** The calculation of the coefficients $RCC_{j}^{C}$ (j=1,2,…,n) of relative closeness to the MIC for each diversification vector $d_{j}$, using the following formula:
  (34) $$RCC_{j}^{C}=\frac{d_{j}^{C^{-}}}{d_{j}^{C^{+}}+d_{j}^{C^{-}}}.$$
**Step 8.** The calculation of the vector of objective criteria weights:
  (35) $$w=\left(w_{1},w_{2},\ldots ,w_{n}\right)$$
  where:
  (36) $$w_{j}=\frac{RCC_{j}^{C}}{\sum_{j=1}^{n}RCC_{j}^{C}}$$
  for j=1, 2,…,n.

**Stage 3:** The extended TOPSIS method for GDM without the aggregation of individual decision matrices.

The developed extended TOPSIS for GDM without the aggregation of individual decision matrices consists of the following steps.

**Step 1.** The normalization, for each decision maker $DM_{k}$ (k=1,2,…,K), of their individual decision matrix, as given by Equation (21), and obtaining the matrix $Y^{k}$, of the form
  (37) $$Y^{k}=\begin{array}{cc} DM_{k} & \begin{array}{cc} \begin{array}{cc} C_{1} & \;\;C_{2}\;\; \end{array} & \begin{array}{cc} \cdots & \;C_{n} \end{array} \end{array}\\ \begin{array}{c} \begin{array}{c} A_{1}\\ A_{2} \end{array}\\ \begin{array}{c} \vdots \\ A_{m} \end{array} \end{array} & \left(\begin{array}{cc} \begin{array}{cc} y_{11}^{k} & y_{12}^{k}\\ y_{21}^{k} & y_{22}^{k} \end{array} & \begin{array}{cc} \cdots & y_{1n}^{k}\\ \cdots & y_{2n}^{k} \end{array}\\ \begin{array}{cc} \vdots & \vdots \\ y_{m1}^{k} & y_{m2}^{k} \end{array} & \begin{array}{cc} \ddots & \vdots \\ \cdots & y_{mn}^{k} \end{array} \end{array}\right) \end{array}$$
  using the following formula for j=1,…,n [38]:
  (38) $$y_{ij}^{k}=\{\begin{array}{ccc} \left[\frac{{\underline{x}}_{ij}^{k}}{\sum_{i=1}^{m}{\bar{x}}_{ij}^{k}},\frac{{\bar{x}}_{ij}^{k}}{\sum_{i=1}^{m}{\underline{x}}_{ij}^{k}}\right] & \mathrm{if} & j\in B\\ \left[\frac{1/{\bar{x}}_{ij}^{k}}{\sum_{i=1}^{m}1/{\underline{x}}_{ij}^{k}},\frac{1/{\underline{x}}_{ij}^{k}}{\sum_{i=1}^{m}1/{\bar{x}}_{ij}^{k}}\right] & \mathrm{if} & j\in C \end{array}.$$

**Remark** **1.** *Note that the normalization method, Equation (38), used above does not provide the property that the normalized elements* $y_{ij}^{k}$ *belong to the interval* [0,1]*. If we require this property to be satisfied, the elements of the matrix* $Y^{k}$ *can be recalculated using the following formula [38]:*

(39) $$z_{ij}^{k}=\left[\frac{{\underline{y}}_{ij}^{k}}{\sqrt{\sum_{i=1}^{m}\left[{\left({\underline{y}}_{ij}^{k}\right)}^{2}+{\left({\bar{y}}_{ij}^{k}\right)}^{2}\right]}},\frac{{\bar{y}}_{ij}^{k}}{\sqrt{\sum_{i=1}^{m}\left[{\left({\underline{y}}_{ij}^{k}\right)}^{2}+{\left({\bar{y}}_{ij}^{k}\right)}^{2}\right]}}\right].$$

*As the final result, we obtain normalized decision matrices* $Z^{k}$ (k=1,2,…,K)*:*

(40) $$Z^{k}=\begin{array}{cc} DM_{k} & \begin{array}{cc} \begin{array}{cc} C_{1} & \;\;C_{2}\;\; \end{array} & \begin{array}{cc} \cdots & \;C_{n}\; \end{array} \end{array}\\ \begin{array}{c} \begin{array}{c} A_{1}\\ A_{2} \end{array}\\ \begin{array}{c} \vdots \\ A_{m} \end{array} \end{array} & \left(\begin{array}{cc} \begin{array}{cc} z_{11}^{k} & z_{12}^{k}\\ z_{21}^{k} & z_{22}^{k} \end{array} & \begin{array}{cc} \cdots & z_{1n}^{k}\\ \cdots & z_{2n}^{k} \end{array}\\ \begin{array}{cc} \vdots & \vdots \\ z_{m1}^{k} & z_{m2}^{k} \end{array} & \begin{array}{cc} \ddots & \vdots \\ \cdots & z_{mn}^{k} \end{array} \end{array}\right) \end{array}$$

**Step 2.** The calculation of the weighted normalized individual matrices $V^{k}$ (k=1,2,…,K):
  (41) $$V^{k}=\begin{array}{cc} DM_{k} & \begin{array}{cc} \begin{array}{cc} C_{1}\; & \;C_{2}\;\; \end{array} & \begin{array}{cc} \cdots & \;C_{n} \end{array} \end{array}\;\\ \begin{array}{c} \begin{array}{c} A_{1}\\ A_{2} \end{array}\\ \begin{array}{c} \vdots \\ A_{m} \end{array} \end{array} & \left(\begin{array}{cc} \begin{array}{cc} v_{11}^{k} & v_{12}^{k}\\ v_{21}^{k} & v_{22}^{k} \end{array} & \begin{array}{cc} \cdots & v_{1n}^{k}\\ \cdots & v_{2n}^{k} \end{array}\\ \begin{array}{cc} \vdots & \vdots \\ v_{m1}^{k} & v_{m2}^{k} \end{array} & \begin{array}{cc} \ddots & \vdots \\ \cdots & v_{mn}^{k} \end{array} \end{array}\right) \end{array}$$
  where:
  (42) $$v_{ij}^{k}=w_{j}z_{ij}^{k}=\left[w_{j}{\underline{z}}_{ij}^{k},w_{j}{\bar{z}}_{ij}^{k}\right]$$
  and $w_{j}$ (j=1,2,…,n) are the objective criteria weights obtained in Stage 2.
**Step 3.** The construction, for each alternative $A_{i}$ (i=1, 2,…,m), of the matrix $A^{i}$:
  (43) $$A^{i}=\begin{array}{cc} A_{i} & \;\begin{array}{cc} \begin{array}{cc} C_{1} & \;C_{2} \end{array} & \begin{array}{cc} \cdots & \;C_{n} \end{array} \end{array}\;\;\\ \begin{array}{c} \begin{array}{c} DM_{1}\\ DM_{2} \end{array}\\ \begin{array}{c} \vdots \\ DM_{K} \end{array} \end{array} & \left(\begin{array}{cc} \begin{array}{cc} v_{i1}^{1} & v_{i2}^{1}\\ v_{i1}^{2} & v_{i2}^{2} \end{array} & \begin{array}{cc} \cdots & v_{in}^{1}\\ \cdots & v_{in}^{2} \end{array}\\ \begin{array}{cc} \vdots & \vdots \\ v_{i1}^{K} & v_{i2}^{K} \end{array} & \begin{array}{cc} \ddots & \vdots \\ \cdots & v_{in}^{K} \end{array} \end{array}\right) \end{array}.$$
**Step 4.** The determination of the PIS $\left(A^{+}\right)$:
  (44) $$A^{+}=\begin{array}{cc} & \begin{array}{cc} \begin{array}{cc} C_{1}\;\; & \;C_{2} \end{array}\;\; & \begin{array}{cc} \cdots & \;C_{n} \end{array} \end{array}\;\;\\ \begin{array}{c} \begin{array}{c} DM_{1}\\ DM_{2} \end{array}\\ \begin{array}{c} \vdots \\ DM_{K} \end{array} \end{array} & \left(\begin{array}{cc} \begin{array}{cc} v_{1}^{1+} & v_{2}^{1+}\\ v_{1}^{2+} & v_{2}^{2+} \end{array} & \begin{array}{cc} \cdots & v_{k}^{1+}\\ \cdots & v_{n}^{2+} \end{array}\\ \begin{array}{cc} \vdots & \vdots \\ v_{1}^{K+} & v_{2}^{K+} \end{array} & \begin{array}{cc} \ddots & \vdots \\ \cdots & v_{n}^{K+} \end{array} \end{array}\right) \end{array}$$
  where $v_{j}^{k+}=\underset{i}{\max}v_{ij}^{k}$ for j=1, 2,…,n and k=1, 2,…,K and of NIS $\left(A^{-}\right)$:
  (45) $$A^{-}=\begin{array}{cc} & \begin{array}{cc} \begin{array}{cc} C_{1}\;\; & \;C_{2} \end{array}\;\; & \begin{array}{cc} \cdots & \;C_{n}\;\; \end{array} \end{array}\\ \begin{array}{c} \begin{array}{c} DM_{1}\\ DM_{2} \end{array}\\ \begin{array}{c} \vdots \\ DM_{K} \end{array} \end{array} & \left(\begin{array}{cc} \begin{array}{cc} v_{1}^{1-} & v_{2}^{1-}\\ v_{1}^{2-} & v_{2}^{2-} \end{array} & \begin{array}{cc} \cdots & v_{k}^{1-}\\ \cdots & v_{n}^{2-} \end{array}\\ \begin{array}{cc} \vdots & \vdots \\ v_{1}^{K-} & v_{2}^{K-} \end{array} & \begin{array}{cc} \ddots & \vdots \\ \cdots & v_{n}^{K-} \end{array} \end{array}\right) \end{array}$$
  where $v_{j}^{k-}=\underset{i}{\min}v_{ij}^{k}$ for j=1,2,…,n and k=1,2,…,K.
**Step 5.** The calculation of the distance of each matrix $A^{i}$, representing the alternative $A_{i}$ (i=1,…,m), from the PIS:
  (46) $$d_{i}^{A+}=\sqrt{\sum_{k=1}^{K}\sum_{j=1}^{n}\left[{\left({\underline{v}}_{ij}^{k}-{\underline{v}}_{j}^{k+}\right)}^{2}+{\left({\bar{v}}_{ij}^{k}-{\bar{v}}_{j}^{k+}\right)}^{2}\right]},$$
  and from the NIS:
  (47) $$d_{i}^{A-}=\sqrt{\sum_{k=1}^{K}\sum_{j=1}^{n}\left[{\left({\underline{v}}_{ij}^{k}-{\underline{v}}_{j}^{k-}\right)}^{2}+{\left({\bar{v}}_{ij}^{k}-{\bar{v}}_{j}^{k-}\right)}^{2}\right]}.$$
**Step 6.** The calculation of the coefficients $RCC_{i}^{A}$ (i=1, 2,…,m) of relative closeness to the PIS for each alternative $A_{i}$ (i=1,…,m), using the following formula:
  (48) $$RCC_{i}^{A}=\frac{d_{i}^{A-}}{d_{i}^{A-}+d_{i}^{A+}}.$$
**Step 7.** The ranking of alternatives in descending order, using $RCC_{i}^{A}$, and the determination of the best one.

## 4. A Numerical Example and Results 

The approach proposed in Section 3 will now be illustrated with a numerical example, taken from [38], related to the evaluation of the authorities of a university in China. The set of alternatives $\left\{A_{1},\;A_{2},\;A_{3}\right\}$ consists of the president and two vice presidents, who are evaluated by teams of teachers, $DM_{1}$, researchers, $DM_{2}$, and undergraduates, $DM_{3}$. The DMs evaluate the presidents with respect to leadership, $C_{1}$, performance, $C_{2}$, and style of work, $C_{3}$, using a point scale from 0 to 100. The team ratings are represented by INs, where the lower end is the minimum and the upper end is the maximum ratings among the group members. The individual decision matrices are presented in Table 1. 

**Figure 3:**
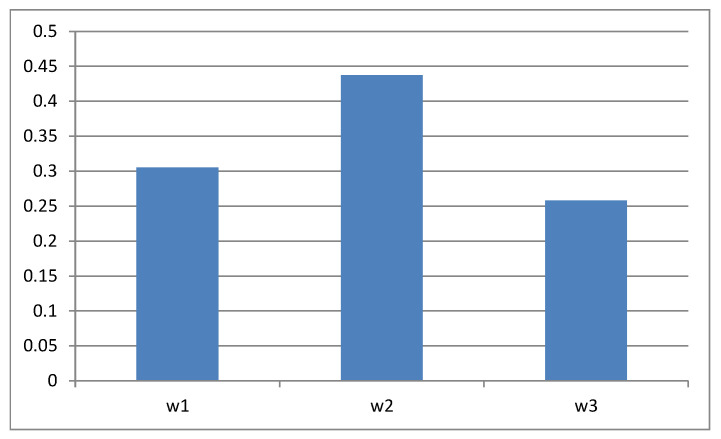
Objective criteria weights.

The first main step of the proposed approach is to determine the objective criteria weights, as described in Stage 2 of Section 3. The individual decision matrices are normalized (see Table 2) and then transformed into matrices of criteria (see Table 3). Next, for each criterion matrix, the entropy and diversification vectors are determined (see Table 4 and Table 5). Using the diversification vectors, we construct a diversification matrix, which is the basis for calculating the objective criteria weights using the interval TOPSIS method. Table 6 presents reference points—in this case, the MIC and LIC. After calculating the distance of each row of the diversification matrix from the MIC and LIC, the RCCs are calculated (see Table 7). These coefficients, after normalization, are the objective criteria weights (see Table 7 and Figure 3). In our example, we obtain the following vector:

w=(0.3049, 0.4372, 0.2579).

**Figure 4:**
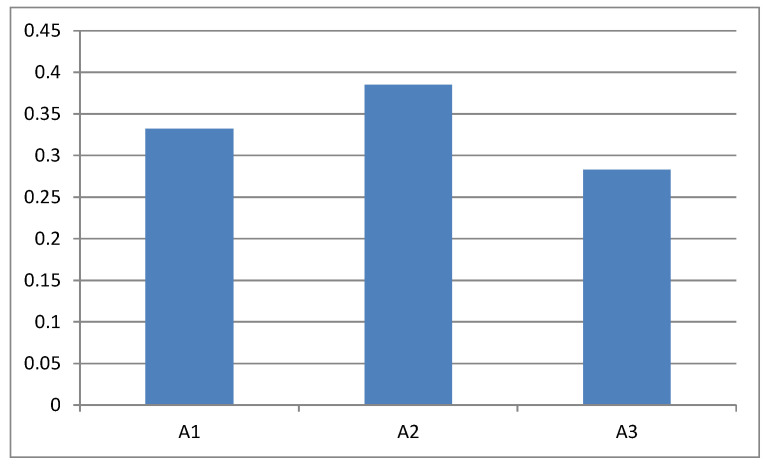
The ranking of the alternatives.

The second main step of the proposed approach is to use an extension of the TOPSIS method for GDM without the aggregation of individual matrices, as described in Stage 3 of Section 3. The individual decision matrices (see Table 1) are normalized (see Table 8) using Equation (38) and then Equation (39). Using objective criteria weights (see Table 7), we calculate the weighted normalized decision matrices (see Table 9). These matrices are the basis for constructing the matrix for each alternative (see Table 10) of the form (43). Now, we apply the extended TOPSIS method for the matrices of alternatives for ranking the alternatives. Table 11 presents reference points—in this case, the PIS and NIS. Finally, the distances of the alternatives from the PIS and NIS and the RCCs are calculated (see Table 12). Based on these coefficients, the ranking of the alternatives is as follows:

$$A_{3}≺A_{1}≺A_{2}$$

where “≺” means “inferior to” (see Table 12 and Figure 4). It means that the highest rating is given to the vice president, $A_{2}$. The symbol J in Table 12 represents the normalized RCCs.

## 5. Comparison of the Proposed Method with Other, Similar Approaches 

In the following, the approach proposed in Section 3 will be compared with other, similar approaches. In practice, the most common methods for GDM use a certain operator to aggregate the individual decision matrices, given by Equation (21), into a group matrix X of the form Equation (7), which is the starting point for the ranking of alternatives. To compare the results obtained by the proposed method (PM), we use the following operators:

- AM—arithmetic mean, defined by:
  $$x_{ij}=\frac{1}{K}\sum_{k=1}^{K}x_{ij}^{k}=\left[\frac{1}{K}\sum_{k=1}^{K}{\underline{x}}_{ij}^{k},\frac{1}{K}\sum_{k=1}^{K}{\bar{x}}_{ij}^{k}\right];$$
- GM—geometric mean, defined by:
  $$x_{ij}={\left(\prod_{k=1}^{K}x_{ij}^{k}\right)}^{\frac{1}{K}}=\left({\left(\prod_{k=1}^{K}{\underline{x}}_{ij}^{k}\right)}^{\frac{1}{K}},{\left(\prod_{k=1}^{K}{\bar{x}}_{ij}^{k}\right)}^{\frac{1}{K}}\right);$$
- WM—weighted mean, defined by:
  $$x_{ij}=\overset{K}{\underset{k=1}{\sum }}\lambda_{k}x_{ij}^{k}=\left(\overset{K}{\underset{k=1}{\sum }}\lambda_{k}{\underline{x}}_{ij}^{k},\overset{K}{\underset{k=1}{\sum }}\lambda_{k}{\bar{x}}_{ij}^{k}\right)$$
  where $\lambda_{k}$ are weights that determine the importance of the DMs, such that $\lambda_{k}\in \left[0,1\right]$ and $\overset{K}{\underset{k=1}{\sum }}\lambda_{k}=1$.

**Figure 5:**
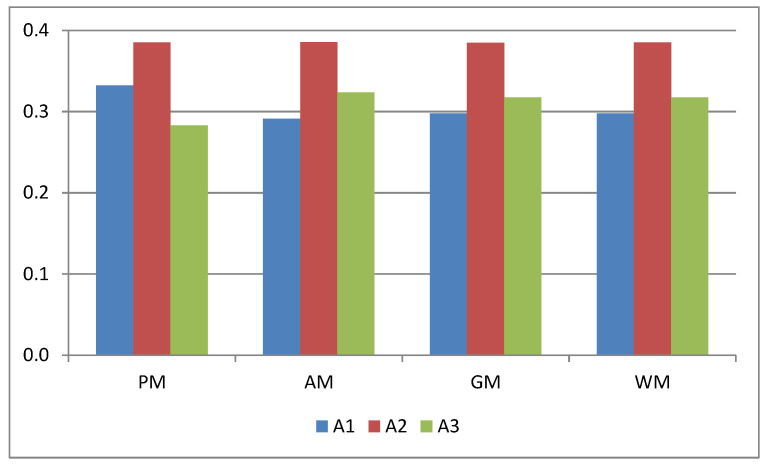
Comparison of results.

In the WM method, the vector of DM weights λ=(0.2661,0.3573,0.3766) is determined by the method proposed by [38]. Next, based on the matrix X, we determine the objective criteria weights using the method proposed by Lotfi and Fallahnejad [35]. In this case, the criteria weights are in the form of INs, so we do not compare them with the criteria weights obtained by the proposed method described in Stage 2 of Section 3 and presented in Table 7. To obtain the ranking of the alternatives, we use the normalization method proposed by Jahanshahloo et al. [27]; the PIS and NIS are determined using Equations (5) and (6), whereas the distances of the alternatives from the PIS and NIS are calculated using Equations (46) and (47), where K=1. Because the analyzed methods are significantly different, to compare the final results we use the indicator J instead of the RRCs. Table 13 and Figure 5 present the results obtained. We can notice that all the analyzed methods indicated alternative $A_{2}$ as the best one, and the obtained values of the indicator J are similar. On the other hand, methods that use an aggregation operator give a different ranking than the proposed method, of the form:

$$A_{1}≺A_{3}≺A_{2}$$

where alternatives $A_{1}$ and $A_{3}$ are swapped.

## 6. Conclusions 

This paper presents a new extension of the TOPSIS method for GDM, using INs. It is an alternative to methods based on the aggregation of individual matrices. It uses the transformation of decision matrices into criteria matrices to determine objective criteria weights, while it uses alternatives matrices to create rankings of alternatives. The numerical example shows that the results obtained by the proposed method differ from the results obtained by the methods based on the aggregation of individual matrices using the arithmetic mean, geometric mean, and weighted mean (with weights reflecting the importance assigned to the DMs). 

However, it is worth noting that the proposed method has some limitations, as it uses data in the form of INs. This implies the necessity of extending the proposed method to other types of imprecise data, which will be the subject of further research. Furthermore, the proposed method should be extended by taking into account the subjective criteria weights and the subjective and objective weights of the DMs, to ensure that all key elements in the decision-making process are taken into account.

## Figures and Tables

**Table 1:** Individual decision matrices.

		*C*_1_	*C*_2_	*C*_3_
$DM_{1}$	$A_{1}$	[60, 90]	[72, 86]	[85, 92]
	$A_{2}$	[77, 81]	[69, 93]	[83, 88]
	$A_{3}$	[80, 96]	[59, 87]	[68, 85]
$DM_{2}$	$A_{1}$	[77, 83]	[68, 86]	[82, 90]
	$A_{2}$	[93, 98]	[76, 86]	[65, 87]
	$A_{3}$	[79, 85]	[72, 92]	[81, 97]
$DM_{3}$	$A_{1}$	[85, 86]	[76, 86]	[80, 97]
	$A_{2}$	[79, 87]	[75, 89]	[81, 93]
	$A_{3}$	[62, 82]	[84, 89]	[78, 82]

**Table 2:** Normalized individual decision matrices for the calculation of criteria weights.

		*C*_1_	*C*_2_	*C*_3_
$DM_{1}$	$A_{1}$	[0.2247, 0.3371]	[0.2707, 0.3233]	[0.3208, 0.3472]
	$A_{2}$	[0.2884, 0.3034]	[0.2594, 0.3496]	[0.3132, 0.3321]
	$A_{3}$	[0.2996, 0.3596]	[0.2218, 0.3271]	[0.2566, 0.3208]
$DM_{2}$	$A_{1}$	[0.2895, 0.3120]	[0.2576, 0.3258]	[0.2993, 0.3285]
	$A_{2}$	[0.3496, 0.3684]	[0.2879, 0.3258]	[0.2372, 0.3175]
	$A_{3}$	[0.2970, 0.3195]	[0.2727, 0.3485]	[0.2956, 0.3540]
$DM_{3}$	$A_{1}$	[0.3333, 0.3373]	[0.2879, 0.3258]	[0.2941, 0.3566]
	$A_{2}$	[0.3098, 0.3412]	[0.2841, 0.3371]	[0.2978, 0.3419]
	$A_{3}$	[0.2431, 0.3216]	[0.3182, 0.3371]	[0.2868, 0.3015]

**Table 3:** Matrices for each criterion.

		*DM*_1_	*DM*_2_	*DM*_3_
$C_{1}$	$A_{1}$	[0.2247, 0.3371]	[0.2895, 0.3120]	[0.3333, 0.3373]
	$A_{2}$	[0.2884, 0.3034]	[0.3496, 0.3684]	[0.3098, 0.3412]
	$A_{3}$	[0.2996, 0.3596]	[0.2970, 0.3195]	[0.2431, 0.3216]
$C_{2}$	$A_{1}$	[0.2707, 0.3233]	[0.2576, 0.3258]	[0.2879, 0.3258]
	$A_{2}$	[0.2594, 0.3496]	[0.2879, 0.3258]	[0.2841, 0.3371]
	$A_{3}$	[0.2218, 0.3271]	[0.2727, 0.3485]	[0.3182, 0.3371]
$C_{3}$	$A_{1}$	[0.3208, 0.3472]	[0.2993, 0.3285]	[0.2941, 0.3566]
	$A_{2}$	[0.3132, 0.3321]	[0.2372, 0.3175]	[0.2978, 0.3419]
	$A_{3}$	[0.2566, 0.3208]	[0.2956, 0.3540]	[0.2868, 0.3015]

**Table 4:** Vectors of entropy.

$e_{1}$	([0.9605, 0.9978], [0.9893, 0.9975], [0.9767, 0.9997])
$e_{2}$	([0.9446, 0.9995], [0.9669, 0.9995], [0.9834, 0.9999])
$e_{3}$	([0.9807, 0.9995], [0.9672, 0.9990], [0.9820, 0.9977])

**Table 5:** Vectors of diversification.

$d_{1}$	([0.0022, 0.0395], [0.0025, 0.0107], [0.0003, 0.0233])
$d_{2}$	([0.0005, 0.0554], [0.0005, 0.0331], [0.0001, 0.0166])
$d_{3}$	([0.0005, 0.0193], [0.0010, 0.0328], [0.0023, 0.0180])

**Table 6:** MIC and LIC.

$C^{+}$	([0.0005, 0.0554], [0.0010, 0.0328], [0.0003, 0.0233])
$C^{-}$	([0, 0], [0, 0], [0, 0])

**Table 7:** Objective criteria weights.

	$d_{j}^{C+}$	$d_{j}^{C-}$	$RRC_{j}^{C}$	$w_{j}$
$C_{1}$	0.0272	0.0472	0.6340	0.3049
$C_{2}$	0.0067	0.0666	0.9091	0.4372
$C_{3}$	0.0365	0.0422	0.5362	0.2579

**Table 8:** Normalized individual decision matrices for the TOPSIS method.

		*C*_1_	*C*_2_	*C*_3_
$DM_{1}$	$A_{1}$	[0.1572, 0.2902]	[0.1876, 0.2980]	[0.2260, 0.2747]
	$A_{2}$	[0.2018, 0.2611]	[0.1798, 0.3223]	[0.2207, 0.2628]
	$A_{3}$	[0.2096, 0.3095]	[0.1537, 0.3015]	[0.1808, 0.2538]
$DM_{2}$	$A_{1}$	[0.2045, 0.2354]	[0.1803, 0.2787]	[0.2098, 0.2768]
	$A_{2}$	[0.2470, 0.2780]	[0.2015, 0.2787]	[0.1663, 0.2676]
	$A_{3}$	[0.2098, 0.2411]	[0.1909, 0.2982]	[0.2073, 0.2983]
$DM_{3}$	$A_{1}$	[0.2348, 0.2681]	[0.2029, 0.2579]	[0.2071, 0.2858]
	$A_{2}$	[0.2183, 0.2712]	[0.2002, 0.2669]	[0.2097, 0.2740]
	$A_{3}$	[0.1713, 0.2556]	[0.2242, 0.2669]	[0.2019, 0.2416]

**Table 9:** Weighted normalized individual decision matrices for the TOPSIS method.

		*C*_1_	*C*_2_	*C*_3_
$DM_{1}$	$A_{1}$	[0.0479, 0.0885]	[0.0820, 0.1303]	[0.0583, 0.0708]
	$A_{2}$	[0.0615, 0.0796]	[0.0786, 0.1409]	[0.0569, 0.0678]
	$A_{3}$	[0.0639, 0.0944]	[0.0672, 0.1318]	[0.0466, 0.0655]
$DM_{2}$	$A_{1}$	[0.0623, 0.0718]	[0.0788, 0.1219]	[0.0541, 0.0714]
	$A_{2}$	[0.0753, 0.0848]	[0.0881, 0.1219]	[0.0429, 0.0690]
	$A_{3}$	[0.0640, 0.0735]	[0.0835, 0.1304]	[0.0535, 0.0769]
$DM_{3}$	$A_{1}$	[0.0716, 0.0817]	[0.0887, 0.1128]	[0.0534, 0.0737]
	$A_{2}$	[0.0666, 0.0827]	[0.0875, 0.1167]	[0.0541, 0.0707]
	$A_{3}$	[0.0522, 0.0779]	[0.0980, 0.1167]	[0.0521, 0.0623]

**Table 10:** Matrices of alternatives.

		*C*_1_	*C*_2_	*C*_3_
$A_{1}$	$DM_{1}$	[0.0479, 0.0885]	[0.0820, 0.1303]	[0.0583, 0.0708]
	$DM_{2}$	[0.0623, 0.0718]	[0.0788, 0.1219]	[0.0541, 0.0714]
	$DM_{3}$	[0.0716, 0.0817]	[0.0887, 0.1128]	[0.0534, 0.0737]
$A_{2}$	$DM_{1}$	[0.0615, 0.0796]	[0.0786, 0.1409]	[0.0569, 0.0678]
	$DM_{2}$	[0.0753, 0.0848]	[0.0881, 0.1219]	[0.0429, 0.0690]
	$DM_{3}$	[0.0666, 0.0827]	[0.0875, 0.1167]	[0.0541, 0.0707]
$A_{3}$	$DM_{1}$	[0.0639, 0.0944]	[0.0672, 0.1318]	[0.0466, 0.0655]
	$DM_{2}$	[0.0640, 0.0735]	[0.0835, 0.1304]	[0.0535, 0.0769]
	$DM_{3}$	[0.0522, 0.0779]	[0.0980, 0.1167]	[0.0521, 0.0623]

**Table 11:** PIS and NIS.

		*C*_1_	*C*_2_	*C*_3_
$A^{+}$	$DM_{1}$	[0.0639, 0.0944]	[0.0786, 0.1409]	[0.0583, 0.0708]
	$DM_{2}$	[0.0753, 0.0848]	[0.0835, 0.1304]	[0.0535, 0.0769]
	$DM_{3}$	[0.0716, 0.0817]	[0.0980, 0.1167]	[0.0534, 0.0737]
$A^{-}$	$DM_{1}$	[0.0479, 0.0885]	[0.0672, 0.1318]	[0.0466, 0.0655]
	$DM_{2}$	[0.0623, 0.0718]	[0.0788, 0.1219]	[0.0429, 0.0690]
	$DM_{3}$	[0.0522, 0.0779]	[0.0887, 0.1128]	[0.0521, 0.0623]

**Table 12:** The ranking of the alternatives—*R*.

	$d_{j}^{A+}$	$d_{j}^{A-}$	$RCC_{j}^{A}$	R	J
$A_{1}$	0.0313	0.0322	0.5076	2	0.3322
$A_{2}$	0.0255	0.0364	0.5884	1	0.3851
$A_{3}$	0.0340	0.0258	0.4318	3	0.2826

**Table 13:** Comparison of results.

	PM	AM	GM	WM
$A_{1}$	0.332244	0.291138	0.297903	0.297472
$A_{2}$	0.385121	0.385427	0.384682	0.385048
$A_{3}$	0.282635	0.323435	0.317416	0.317480

## Data Availability

Not applicable.

## Abbreviations

TOPSIS	Technique for Order Preference by Similarity to Ideal Solution
GDM	Group Decision Making
IN	Interval Number
DM	Decision Maker
MCDM	Multiple Criteria Decision Making
PIS	Positive Ideal Solution
NIS	Negative Ideal Solution
RCC	Relative Closeness Coefficient
MCGDM	Multiple Criteria Group Decision Making
MIC	Most Important Criterion
LIC	Least Important Criterion
PM	Proposed Method
AM	Arithmetic Mean
GM	Geometric Mean
WM	Weighted Mean
